# Post-crash management of road traffic injury victims in Iran. Stakeholders' views on current barriers and potential facilitators

**DOI:** 10.1186/1471-227X-9-8

**Published:** 2009-05-12

**Authors:** Davoud Khorasani-Zavareh, Hamid Reza Khankeh, Reza Mohammadi, Lucie Laflamme, Ali Bikmoradi, Bo JA Haglund

**Affiliations:** 1 Division of Social Medicine, Department of Public Health Sciences, Karolinska Institutet, Norrbacka, SE-171 76 Stockholm, Sweden; 2 National Public Health Management Centre (NPMC), Tabriz University of Medical Sciences, Tabriz, Islamic Republic of Iran; 3 Urmia University of Medical Sciences, Oroumiyeh, Islamic Republic of Iran; 4 Department of Nursing, University of Social Welfare and Rehabilitation, Tehran, Islamic Republic of Iran; 5 Division of International Health Care Research (IHCAR), Department of Public Health Sciences, Karolinska Institutet, SE-171 77 Stockholm, Sweden; 6 Medical Management Centre, Department of LIME, Karolinska Institutet, 171 77 Stockholm, Sweden; 7 Hamadan University of Medical Sciences, Hamadan, Islamic Republic of Iran

## Abstract

**Background:**

Road traffic injuries are a major public health problem, especially in low- and middle-income countries. Post-crash management can play a significant role in minimizing crash consequences and saving lives. Iran has one of the highest mortality rates from road traffic injuries in the world. The present study attempts to fill the knowledge gap and explores stakeholders' perceptions of barriers to – and facilitators of – effective post-crash management in Iranian regions.

**Methods:**

Thirty-six semi-structured interviews were conducted with medical services personnel, police officers, members of Red Crescent, firefighters, public-health professionals, road administrators; some road users and traffic injury victims. A qualitative approach using grounded theory method was employed to analyze the material gathered.

**Results:**

The core variable was identified as "poor quality of post crash management". Barriers to effective post-crash management were identified as: involvement of laypeople; lack of coordination; inadequate pre-hospital services; shortcomings in infrastructure. Suggestions for laypeople included: 1) a public education campaign in first aid, the role of the emergency services, cooperation of the public at the crash site, and 2) target-group training for professional drivers, police officers and volunteers involved at the crash scene. An integrated trauma system and infrastructure improvement also is crucial to be considered for effective post-crash management.

**Conclusion:**

To sum up, it seems that the involvement of laypeople could be a key factor in making post-crash management more effective. But system improvements are also crucial, including the integration of the trauma system and its development in terms of human resources (staffing and training) and physical resources as well as the infrastructure development.

## Background

Road traffic injuries (RTIs) are a major public health problem, requiring concerted efforts [1], in the fields of both crash prevention and post-crash management (PCM). It is often possible to minimize crash consequences by promptly providing effective pre-hospital services [2-8]. Indeed, each year, many of the 1.2 million lives lost could be saved and much of the ensuing disability suffered by the 50 million injured could be prevented if rapid and competent pre-hospital services were available at the crash scene [1,9]. But, in many countries, few victims receive treatment at the crash scene and fewer still can hope to be transported to hospital by ambulance, promptly or at all [10]. Transport, when available, is usually provided by untrained people; e.g., relatives, taxi drivers, truck drivers, or by police officers [11,12]. Conversely, in spite of it contributing to saving lives or reducing consequences, the involvement of untrained people at the crash scene may engender serious neurological injuries, severe sequels or fatal consequences occasioned either when extricating victims from vehicles or when transporting them without adequate immobilization [11,13-15].

PCM leaves much to be desired especially – but not exclusively – in low-and middle-income countries [11,12,16]. In Iran, according to a national survey from 2003, only 14% of RTI victims were transported by ambulance and 10% were rescued by trained personnel [17,18]. As road traffic injuries are a major cause of death in the country[17,19,20], substantial efforts have been made in recent years to improve Emergency Medical Services [21], not only by increasing the number of ambulances and ambulance dispatch sites but also by providing better equipment, more staff, and educational plans for emergency team members. Whether additional and context-relevant efforts are needed is uncertain, however, and how to move forward is unclear.

Studies in the field performed in settings other than Iran have used mainly quantitative designs that have helped to quantify needs more than to obtain new perspectives. The few in-depth qualitative studies available [22-27] have focused on specific groups of road users and were limited in scope. These studies do not provide much information as to various stakeholders' perceptions regarding how PCM can be made more effective. But stakeholders' perceptions are indeed important for quality improvement [28-33]. The present study attempts to fill this knowledge gap and explores stakeholders' perceptions of barriers to – and facilitators of – effective PCM in Iranian regions.

## Method

The study was performed using Grounded Theory, which is a suitable method when new areas are to be investigated in an explorative manner or if it has been decided to explore a known area from a fresh perspective [32,34,35].

### Setting

Focus was placed on PCM for road traffic injuries that occurred in West Azarbaijan Province (WAP) and Tehran; both local and national stakeholders were interviewed. The province covers an area of 37,411 km^2^, and the population density is 77 inhabitants/km^2^. In 2005, the total number of fatal road traffic injuries reached 1,018, i.e., 34 per 100,000 of the population [19].

In Iran, Emergency Medical Services, the police and the fire brigades can all function as emergency services and take care of victim management when road traffic crashes occur. People can contact them by dialling the three-digit numbers 115, 110 and 125, respectively. Additional potential actors include different ambulance services and the Red Crescent, whose activities are primarily focused on rendering relief to the victims of natural and man-made disasters in general [36] but who can become involved in multiple crashes and road-victim management. Further, as mentioned earlier, members form public, who are usually present at crash scenes, can also help victims and transport them to hospital.

### Participants

Stakeholders of varying experience and knowledge, representing different perspectives on PCM, were approached for interviews. A few victims were also interviewed because of the unique perspective they could add on what takes place at the crash scene. The number of participants was determined based on saturation principles [34]. From thirty-eight approached participants, thirty-six agreed to be interviewed, including seven members of the Emergency Medical Services (abbreviated as EMS) – physicians, nurses, and technicians -, six police officers (PO), two members of Red Crescent (RC), one firefighter (FF), five public health professionals (PH), two experts members from the Ministry of Road (RT), four experts from Road & Transportation Office (RT), two motorcyclists (MC), two car drivers (CD), and five road traffic injury victims (VI). EMS members and public health professionals had academic knowledge and experience of victim management with regard to training and re-training in their field. Red Crescent members, firefighters and police officers also had experience and training in the field of RTI victim management. Their age range was between 20 and 65, with education level ranging from nine-year intermediate school to professional education in the field of medicine.

### Data collection

Data were gathered through semi-structured interviews beginning with general questions, gradually progressing to more specific ones. Probing was performed according to the reflections of each participant, concerning prior experiences of the post-crash event; perception about barriers to PCM; opinions about facilitators of effective PCM; opinions relating to the role of laypeople at crash scenes; opinions about the organization and coordination of PCM. The interviews lasted between 45 and 80 minutes. They were conducted between March and December 2007.

Finding and contacting eligible participants was made easier by the fact that the principal investigator had experience in the domain, having worked with the management of road traffic injury victims. The interviews were conducted in Persian, transcribed verbatim and summarized in English, and two interviews had read by a researcher who was not a Persian speaker. The rest of the researchers were fluent in both Persian and English.

### Data analysis

The interview transcriptions were compared with the recorded digital files for accuracy. The data gathered were analyzed following the principals put forward by Strauss and Corbin [34,35], i.e., data collection and data analysis took place simultaneously in order to identify ideas, which then guided the next interview. For that purpose, the principal investigator carefully read the whole text to get an impression and obtain an overall understanding of each interview. During the open coding phase, all the interviews were read several times, and key words or phrases, incidents and facts in the text were noted. During this phase, primary codes were extracted. The codes and data were compared for similarities and differences, and then categories and sub-categories were developed. From the first interview, a preliminary set of codes, categories and sub-categories was created and approved by the co-authors and research group. For the first four interviews, the principal investigator came back to the participants and checked the transcription (member check) and the summary of understanding of the interview. Selection of the participants was guided by theoretical sampling method. This process was continued until saturation of each concept was reached and further data collection failed to contribute new information. Accordingly, during interviews, any identified concepts were discussed until saturation. Data saturation supported the sample size. After axial coding, and at the end of the selective coding phase, a core variable was identified.

### Ethical considerations

The study was approved by the National Ethics Committee of Iran [37]. Interviewees were informed that their participation was confidential, anonymous, and voluntary. Information explaining the aim of the study was provided orally and in writing. The interviewees then signed an informed consent form or verbally consented to participate in the study, which included both being interviewed and recorded.

## Results

"Poor quality of post crash management" was the core variable, which mirrored the general views expressed by the participants[38]. Four main barriers (involvement of laypeople; lack of coordination; inadequate pre-hospital services; and shortcomings in infrastructure) and four facilitators (public education campaign; target group training; integrated trauma system; and infrastructure improvement) of effective PCM were identified.

### Barriers

#### Involvement of laypeople

**Figure 1:**
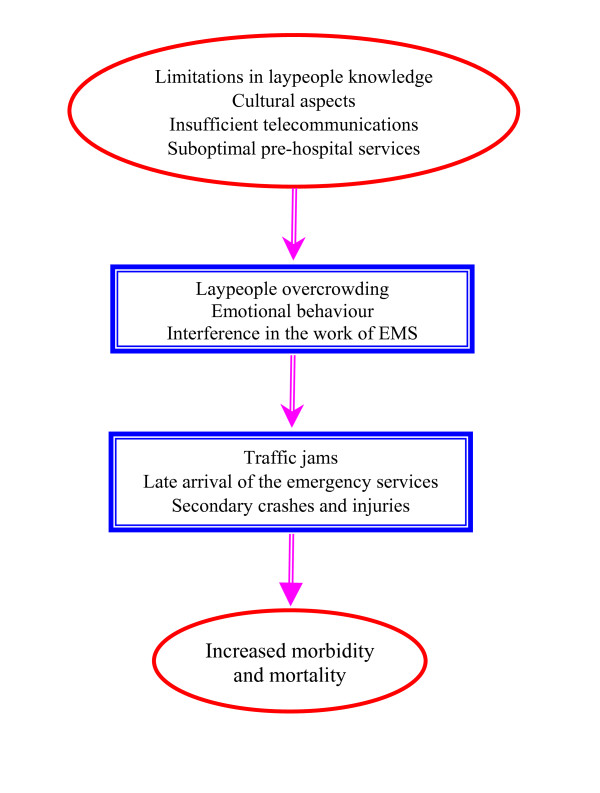
**Conceptual model in barriers related to effective laypeople interaction in post-crash management in Iran**.

The involvement of untrained laypeople as a potential barrier during post-crash events was mentioned by all participants. The main factors identified concerned cultural background, limitations in knowledge and late arrival of the emergency services. Altogether, these factors explain why laypeople gather at a crash scene, the negative effect of their role in rescue activities and how they have the potential to indirectly increase injury morbidity and mortality. Figure 1 show barriers related to the role of laypeople when crashes occur.

The cultural background factors relating to laypeople's involvement that were raised during the interviews included: a willingness to help, humanitarian assistance, individual curiosity, people's sense of haste, excitement, and disorganized cooperation (leading to a crowded crash scene and poor coordination). It was also mentioned that laypeople feel that removing victims from the crash scene and taking them to hospital quickly is better for the victims. Laypeople's limited knowledge related to: how to interact at a crash scene; what information needs to be given to the emergency service; how to use different emergency numbers; and how to provide first aid. Whereas all participants commented on the above, professionals in the EMS and police officers pointed out that such knowledge limitations affected the quality of the information provided (incomplete or wrong) to the emergency services. Police officers, representatives from the Road & Transportation Office and some victims added that laypeople's worries about whether ambulances would arrive on time also influenced the quality of the interactions.

*(EMS/1)People want to help casualties, but they usually don't know first aid, aren't sure what to do before the ambulance arrives and what kind of detailed information they need to give the emergency services when they call them. This can lead to incorrect phone-calls and the wrong information being conveyed*.

Invariably, laypeople are the first to arrive at a crash site. According to most stakeholders, laypeople are often stressed and can easily interfere with the activities of ambulance personnel. They usually remove victims too quickly and take them to hospital in their vehicles. Their involvement is regarded as necessary to alert the emergency services and seen as useful in rural and remote areas. However, members of the EMS and police officers consider that laypeople, when too involved in crashes occurring in urban areas, may easily contribute to wasted time, hamper the emergency services, cause secondary injuries to victims and even provoke new crashes.

*(PO/3) A common problem at crash scenes is the gathering of too many people and their emotional behaviour, which could at worst lead to new crashes and new injuries to the victims. (EMS/2) An example of the latter might be potential spinal cord injuries caused by the victim being moved too fast*.

#### Lack of coordination

Different opinions were gathered concerning crash management and delayed victim transport. At many crashes, the police must be present to take statements, which is important for insurance and legal purposes. According to EMS members, this task wastes precious time and delays the transportation of victims to hospital. Members of other organizations stated that an insufficient number of ambulance dispatch sites could also result in delayed transportation. Moreover, the fact that rescue activities are designed in different ways in different organizations could contribute to delays. Additional factors seen as likely to impede coordination were: lack of a systematic approach to PCM; different ambulance dispatch site locations; existence of parallel organizations with the same activity (different ambulances in the EMS, Red Crescent, hospitals, fire services, private ambulances and some military ambulances); substandard telecommunication equipment; and undeveloped satellite navigation (GPS), which might hamper coordination and cooperation among the organizations.

*(EMS/2) Most of the calls made by laypeople to the emergency services or other organizations concern crashes without casualties. We dispatch our ambulance but since there are no casualties, it is a waste of both time and resources*...

#### Inadequate pre-hospital services

The low number of ambulance dispatch sites is viewed as a hindrance to effective pre-hospital services, together with inadequate human resources (staffing and adequate formal training) and insufficient physical resources (ambulances and their equipment). Some participants stated that the role of a rescuer in post-crash events of road traffic injuries is not clear, and is established differently in different organizations. There is often a lack of police officers at many crash scenes and their lack of crash scene management skills was also seen as a hindrance to effective PCM.

*(PO/3)The fire services are officially responsible for rescue activities particularly in urban area, but they cannot be present at all crashes. However, EMS ambulances usually don't have enough rescue equipment and in some cases, crash victims are trapped in their cars, resulting in the EMS having to call the fire services to come and cut them out*.

#### Shortcomings in infrastructure

Some participants in EMS, public health organizations and police officers stated that shortcomings in the infrastructure constitute an important barrier to effective PCM. These include poor urban infrastructure and no satellite navigation (GPS) or well-established telecommunication systems.

*(EMS/5) There is not usually a traffic lane reserved for the emergency service, and we are stuck in a traffic jam... (RT/2) there are different emergency numbers for organizations and people will be confused over which number they should call when they see a crash and an emergency situation*.

### Facilitators

#### Public education campaign

All participants stated that public education plays an important role in effective PCM and considered it should be widely spread. It was mentioned that, in recent years, many activities and public education campaigns have been implemented by the police, focusing on the primary and secondary prevention of road traffic injuries, but the need for more public education regarding PCM was clearly stressed. It was considered that this should incorporate aspects related to better cooperation of people with the emergency services, basic first aid techniques, the role of the different emergency services in road traffic crashes, as well as safe victim transportation when ambulances are not available. All participants pointed out that the mass media, especially television, were relevant channels of public health information in the Iranian context. On the other hand, it was strongly felt that, since many people still do not have enough knowledge of first aid, their cooperation should be limited to protection of the crash scene and alerting the emergency services, especially at urban crash sites. Their cooperation should also be under the supervision of members of the emergency services.

*(PO/1) People need to learn that they should leave the crash scene immediately when the ambulance team arrives. (EMS/4) Public education is necessary for first aid, recognizing emergency needs, helping the ambulance arrive faster and leaving the crash location carefully and calmly. (PO/1) They should know how to use the different emergency numbers to call the appropriate emergency service*.

#### Target-group training

Most participants stated that training of those who arrive initially at the crash scene was another way of improving crash scene management. The fact that, in Iranian culture, those who help others are held in high esteem, can be beneficial when it comes to PCM. It was proposed that training should include basic principles of safe rescue, Cardio Pulmonary Resuscitation (CPR), victim triage and safe transportation to medical centres. This group could be made up of professional drivers. The same suggestion was made with regard to other people who volunteer their help. Providing a kit of simple equipment and supplies and a special uniform for this group could improve their cooperation. Some participants also recommended an ongoing pilot programme, in which police officers receive special training on how to manage crash sites.

*(RT/2)If we can train some professional drivers (bus drivers, truck drivers, etc.) and if we give them a uniform to show that they are responsible for emergency services as well as some supplies, this might improve crash scene management. (PO/2) Such people are often first on the crash scene, arriving sooner than all other organizations, and if they know first aid and preliminary crash scene management, they will be of more help to the victims*.

#### Integrated trauma system

Combination of rescue activities and the introduction of one emergency telephone number were suggested by most participants. Further, better coordination among organizations was regarded as necessary for effective victim management. It was proposed that all EMS ambulances and Red Crescent ambulances should be equipped with rescue equipment, as well as other vital equipment. Moreover, in order to improve victim rescue, staff training was seen as more important than physical equipment, including the number of ambulances and ambulance dispatch sites. One suggestion for interurban roads was the establishment of a collaborative group consisting of ambulance team members, Red Crescent personnel, police officers and road & transportation officers, which would be more useful in crash black spots. Access to a helicopter ambulance in crowded cities was also regarded as necessary. Both these last two suggestions are currently being implemented in many cities and need to be expanded.

*(EMS/2) Should a system be formulated to do the tasks of police, firefighters, medical staff and rescue teams, all together, (if so) the provision of services would be much better... (FF) if emergency services can have a single emergency number for all calls, it could ease coordination and speed up arrival on the crash scene*.

#### Infrastructure improvement

(Suggestions for improvements to infrastructure were put forward but as part of a long-term strategy. These included better urban infrastructure including establishment of GPS and better telecommunications, including an improved emergency telephone service.

## Discussion

The aims of PCM are to avoid preventable death and disability, to limit the severity of injury and the suffering caused by it, and to ensure the crash survivor's best possible recovery and reintegration into society [1]. Conducted in the Iranian context, our study highlights significant barriers to the achievement of those aims, including laypeople's involvement (in particular in urban settings), suboptimal pre-hospital services and poor coordination among organizations.

### Untrained laypeople's involvement – education

One of the most common issues raised in relation to PCM was the interaction of untrained laypeople and their lack of knowledge and skills in handling the situation in general; and the victims in particular. According to the World Health Organization (WHO) [39], the role of laypeople who are present at a crash scene should be: to contact the emergency services; help to put out fires; and take action to secure the crash scene (e.g. preventing further crashes, preventing harm to rescuers and bystanders, controlling the crowd of onlookers, and applying first aid). It seems that some – but not all – of these WHO recommendations are not fully followed in the study area. More specifically, laypeople extricate – or try to extricate – victims instead of taking action to secure the scene. This might be related in part to the sense of haste and urgency that they also have reported, but also to the late arrival of the emergency services at the scene, which has an adverse effect on the management of the crash scene.

This, in turn, calls for better public information concerning what should preferably be done by laypeople at the crash scene (including calling the emergency service, and not moving any victims unless trained in doing so). Such information should also point out the important role that trained laypeople can play when, among other things, applying first aid e.g., checking the victims' airways, bleeding and circulation [40], and being involved in the scene management. Public education should also emphasize the issue of the emotional behaviour of laypeople and how this can impede the work of ambulance team members, which would be important to address in a public education campaign.

An additional educational aspect to be dealt with is the training of target groups. Indeed, studies from low-and middle-income countries indicate that basic first-aid training for professional drivers (taxi, bus or truck drivers) could help improve PCM, as they can often provide care and transportation [12,41,42]. This could even apply to the combination of formal training of both paramedics, and basic training for laypeople, and the provision of some basic supplies and equipment which could decrease the mortality rate to an even greater extent [43].

### Poor coordination

According to Nathens et al. [44], the trauma system of a given region or country represents a local solution to a complex organizational problem involving the coordination of resources and services provided by many actors and is largely dependent on tradition rather than outcome-driven data. Pre-hospital services (i.e. extrication of trapped casualties in road traffic crashes and their transportation) require coordination of rescue activities by different organizations and groups. Without it, extrication becomes slow, frustrating, and may be dangerous for both victims and rescuers[6]. Lack of coordination as a major barrier to effective PCM has also been raised in earlier studies in case of disaster [45,46]. Bazzoli [2] poses that the most important strategies to counteract this problem include broad-based participation of key stakeholders and changes in trauma delivery. Although various parameters can come into play [42], the study participants mainly referred to difficulties in coordination rather than in equipment, staffing and physical resources.

### Suboptimal pre-hospital services

The vast majority of road traffic deaths in low-and middle-income countries [39,47] and in Iran [17,48] occur in the pre-hospital phase. It has been hypothesized that the reduction in the proportion killed of all those who are involved in road traffic injuries is, at least in part, attributable to an improved provision of emergency medical services[7]. As proposed elsewhere, comprehensive trauma systems [49] should be widely put into place and, according to Zargar et al. [50], they are a must in Iran.

Although rapid improvements in pre-hospital care services have occurred in the country [21], it seems that their administration needs further improvement. Moreover, a holistic approach to the trauma system as a whole might be required. It ought to be underlined that, in rural areas, most of pre-hospital service problems originate from a lack of ambulance dispatch sites and equipment which leads to late arrival of the ambulances, a result that is in line with findings from Mock et al. [42,49].

### Strengths and limitations

This interview-based study gathers the opinions of various actors relating to the barriers to and possible facilitators of effective PCM in the Iranian context. As such, it is one of the few studies adopting a qualitative approach to highlight ways of improving the current situation. The results point to a number of crucial areas in need of improvement, and for which some strategies have been proposed.

As is the case in qualitative studies, the number of participants was relatively small, but all stakeholders were experienced and knowledgeable and saturation was reached. The data was even validated using constant comparison analysis, which means returning to the data in order to verify and develop the categories further. In this vein, the input from previous victims can be regarded as an important contribution – and an originality of the study.

Because of our design, the generalizability of these data is not self-evident. It would definitely be of interest, however, to see the extent to which future research in the same field, but from other parts of the country, yielded similar result.

## Conclusion

Improving PCM helps reduce deaths, disability and the severity of road traffic injuries. The study sheds light on important barriers to effective PCM that need to be tackled in the Iranian context, including the involvement of laypeople, insufficient pre-hospital services and poor coordination. Among other recommendations for laypeople in general, the suggestions gathered include public education campaigns, covering the use of emergency numbers, the role of organizations in crash site management, first aid, better cooperation between laypeople and ambulance personnel and other organizations, and preliminary management of the crash scene before the arrival of the ambulance. Police officers and professional drivers are also an important target group, as their role can be very influential in crash scene management. Their training could cover management of the crash scene, including securing the scene to prevent new crashes and applying first aid for victims before the ambulance arrives, triage of the victims as well as their safe transportation.

Despite improvements in pre-hospital care, the upgrading and improvement of physical resources should also be considered, including improvement in ambulance dispatch sites and their equipment as well as staff. More focus needs to be put on their training and skills. Supplying all ambulances with rescue equipment is strongly recommended. Moreover, improvements to telecommunication systems need to be seriously considered. Furthermore, instead of different pre-hospital services, an integrated trauma system should be considered as a long-term strategy with a focus on research.

## Competing interests

The authors declare that they have no competing interests.

## Authors' contributions

DKZ has made substantial contributions to the conception and design of the study, and taken responsibility for and coordinated the acquisition of data, which he gathered and analyzed. He took part actively in the analysis of the data, in its abstraction and in the writing-up of the manuscript. HRK and LL contributed to the conception and design of the study. HRK was involved in the data collection process and took an active part in the data analysis and results interpretation. LL also took part in the writing-up and finalisation of the manuscript. RM, AB and BH contributed to the study design, data acquisition, results interpretation and writing-up of the manuscript. All authors read and approved the final manuscript.

## Pre-publication history

The pre-publication history for this paper can be accessed here:


